# Protective Effects of Gomisin N against Hepatic Cannabinoid Type 1 Receptor-Induced Insulin Resistance and Gluconeogenesis

**DOI:** 10.3390/ijms19040968

**Published:** 2018-03-23

**Authors:** Arulkumar Nagappan, Dae Young Jung, Ji-Hyun Kim, Myeong Ho Jung

**Affiliations:** 1 Division of Longevity and Biofunctional Medicine, School of Korean Medicine, Pusan National University, Yangsan 50612, Korea; arulbiotechtnau@gmail.com (A.N.); dyjung999@naver.com (D.Y.J.); kimji77@pusan.ac.kr (J.-H.K.); 2 Healthy Aging Korean Medical Research Center, School of Korean Medicine, Pusan National University, Yangsan 50612, Korea

**Keywords:** cannabinoid type 1 receptor, endoplasmic reticulum stress, gluconeogenesis, gomisin N, lipogenesis, insulin resistance

## Abstract

Activation of the hepatic cannabinoid type 1 receptor (CB1R) induces insulin resistance and gluconeogenesis via endoplasmic reticulum (ER) stress, thereby contributing to hyperglycemia. Gomisin N (GN) is a phytochemical derived from *Schisandra chinensis*. In the current study, we investigated the inhibitory effects of GN on hepatic CB1R-mediated insulin resistance and gluconeogenesis in 2-arachidonoylglycerol (AG; an agonist of CB1R)-treated HepG2 cells and in high-fat diet (HFD)-induced obese mice. Treatment with 2-AG induced the expression of ER stress markers, serine/threonine phosphatase *PHLPP1*, *Lipin1*, and ceramide synthesis genes, but reduced the expression of ceramide degradation genes in HepG2 cells. However, GN reversed 2-AG-mediated effects and improved the 2-AG-mediated impairment of insulin signaling. Furthermore, GN inhibited 2-AG-induced intracellular triglyceride accumulation and glucose production in HepG2 cells by downregulation of lipogenesis and gluconeogenesis genes, respectively. In vivo, GN administration to HFD obese mice reduced the HFD-induced increase in fasting blood glucose and insulin levels, which was accompanied with downregulation of HFD-induced expression of CB1R, ER stress markers, ceramide synthesis gene, and gluconeogenesis genes in the livers of HFD obese mice. These findings demonstrate that GN protects against hepatic CB1-mediated impairment of insulin signaling and gluconeogenesis, thereby contributing to the amelioration of hyperglycemia.

## 1. Introduction

The liver plays a major role in maintaining normal blood glucose levels by regulating de novo glucose production and glycogen breakdown. Hepatic glucose production (gluconeogenesis) is essential for the supply of glucose as an energy source to other tissues. However, excessive hepatic gluconeogenesis causes hyperglycemia. Insulin resistance is defined as the disability of insulin to regulate glucose and lipid metabolism in peripheral tissues even at elevated insulin levels in the blood. Hepatic insulin resistance results in the elevation of hepatic glucose production and triglyceride (TG) accumulation by impairing insulin-mediated inhibition of gluconeogenesis and regulating insulin-mediated TG metabolism, respectively, which contributes to hyperglycemia and dyslipidemia [1]. Therefore, the control of hepatic insulin resistance is an attractive therapeutic target for treating type 2 diabetes and hepatic steatosis.

Endogenous cannabinoids, such as arachidonoyl ethanolamide (AEA) and 2-arachidonoylglycerol (2-AG), are bioactive lipid mediators that interact with the cannabinoid type 1 receptor (CB1R) and cannabinoid type 2 receptor (CB2R), and regulate numerous biochemical responses [2]. CB1R is predominantly present in the brain and controls food behavior and energy expenditure [2]. The activation of CB1R in the central nervous system facilitates food intake by modulating the release of orexigenic and anorexigenic neuropeptides in hypothalamic neurons. CB1R is also found in peripheral tissues and controls glucose and lipid metabolism [2]. Activation of hepatic CB1R induces insulin resistance through several mechanisms in the endoplasmic reticulum (ER) stress-dependent manner [3,4,5]. Activation of CB1R inhibits insulin signaling by elevating inhibitory serine-307 phosphorylation of insulin receptor substrate 1 (IRS1) and by stimulating the dephosphorylation of insulin-activated protein kinase B (PKB/AKT) through upregulation of the S/T phosphatase PH domain and leucine-rich repeats protein phosphatase 1 (*PHLPP1*) [3]. Furthermore, it stimulates the expression of *Lipin1*, a phosphatidic acid phosphatase, via ER stress-inducible transcription factor, cAMP-responsive element-binding protein H (CREBH) [4]. This subsequently leads to accumulation of diacylglycerol (DAG), resulting in the phosphorylation of protein kinase C with inhibition of insulin receptor signaling [4]. In addition, CB1R-mediated ER stress increases the production of ceramide by upregulation of de novo ceramide synthesis and downregulation of ceramide degradation, which inhibits insulin signaling [5]. Moreover, hepatic CB1R activation stimulates the lipogenesis transcription factor, sterol regulatory element-binding transcription factor 1c (SREBP1c), and results in increased TG accumulation by upregulating the expression of its downstream lipogenesis genes, including fatty acid synthase (*FAS*), stearoyl-Coenzyme A desaturase 1 (*SCD1*), and acetyl-CoA carboxylase (*ACC*), which contributes to insulin resistance and steatosis [6]. Furthermore, CB1R also increases the expression of gluconeogenesis genes via CREBH and results in the elevated glucose production [7]. Thus, activation of hepatic CB1R plays a role in the development of insulin resistance, type 2 diabetes, and hepatic steatosis [8]. In this regard, selective inhibition of hepatic CB1R signaling could be a potential molecular target for the treatment of type 2 diabetes and hepatic steatosis.

*Schisandra chinensis* has been used as a traditional herbal medicine in Asian countries, such as China, Korea, Japan, and Russia. It has diverse pharmacological activities, including anti-oxidant, anti-tumor, anti-obesity, anti-inflammatory, cardioprotective, and hepatoprotective effects [9]. Recently, we reported that *S. chinensis* has a protective effect against ER stress-induced hepatic steatosis [10]. Gomisin N (GN), a lignan derived from *S. chinensis*, possesses hepatoprotective, anti-cancer, and anti-inflammatory effects [11]. Recently, we reported that GN exerts protective effects against obesity-induced hepatic steatosis and hyperglycemia through inhibition of ER stress and AMP-activated protein kinase (AMPK) activation, respectively [12,13]. As activation of hepatic CB1R signaling has been implicated in the development of insulin resistance, hyperglycemia, and hepatic steatosis, targeted inhibition of hepatic CB1R signaling might provide therapeutic approaches to restore insulin receptor signaling and improve hyperglycemia and hepatic steatosis.

Therefore, in the current study, we investigated the inhibitory effect of GN on hepatic CB1R and CB1R-mediated insulin resistance and gluconeogenesis in 2-arachidonoylglycerol (AG; an agonist of CB1R)-treated HepG2 cells and in high-fat diet (HFD)-induced obese mice. 

## 2. Results

### 2.1. GN Inhibited 2-AG-Induced ER Stress in HepG2 Cells

**Figure 1:**
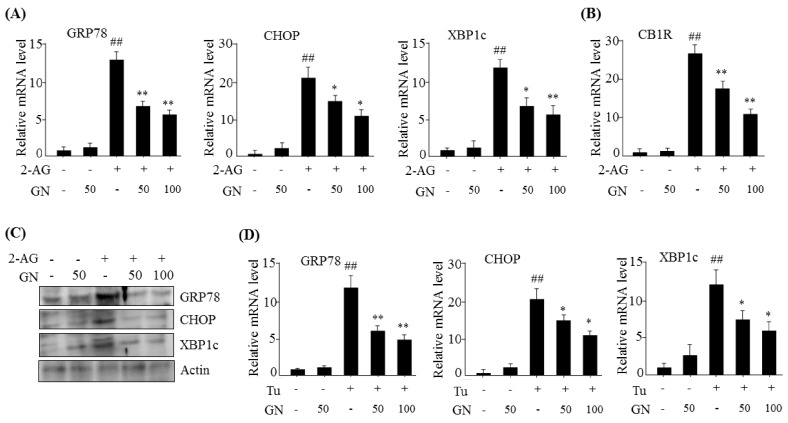
Gomisin N (GN) inhibited 2-AG-induced endoplasmic reticulum (ER) stress in HepG2 cells. HepG2 cells were incubated with 2-AG in the absence or presence of GN (50 or 100 µM) for 12 h. (**A**) qPCR analysis of *GRP78*, *CHOP*, and *XBP1c*; (**B**) qPCR analysis of *CB1R*; (**C**) Western blot analysis of GRP78, CHOP, and XBP1c; (**D**) HepG2 cells were incubated with tunicamycin (Tu) in the absence or presence of GN (50 or 100 µM) for 12 h. qPCR analysis of *GRP78*, *CHOP*, and *XBP1c*. Values are expressed as mean ± SEM (*n* = 3 independent experiments). ^##^ *p* < 0.01 vs. untreated control. * *p* < 0.05, ** *p* < 0.01 vs. 2-AG or tunicamycin-treated control.

The 3-(4,5-dimethylthiazol-2-yl)-2,5-diphenyltetrazolium bromide assay revealed that GN had no cytotoxic effect on HepG2 cells at a concentration of 100 µM. Activation of hepatic CB1R induces ER stress, which contributes to insulin resistance and gluconeogenesis [3,4,5]. Therefore, we first investigated whether GN inhibited CB1R-induced ER stress in HepG2 cells. HepG2 cells were incubated with 2-AG in the absence or presence of different concentrations of GN for 12 h. Treatment with 2-AG increased mRNA level of ER stress markers, including glucose-regulated protein 78 (*GRP78*), C/EBP homologous protein (*CHOP*), and X box-binding protein 1c (*XBP1c*) (Figure 1A), as well as *CB1R* mRNA level (Figure 1B). However, GN treatment suppressed this increase in mRNA levels in a dose-dependent manner. Western blot analysis also showed that 2-AG treatment increased the protein levels of GRP78, CHOP, and XBP1c, but GN treatment reduced the 2-AG-induced increased in ER stress marker levels (Figure 1C), which was consistent with decrease in mRNA levels. Furthermore, to confirm the inhibitory effect of GN on ER stress, we examined the expression of ER stress markers in HepG2 cells treated with tunicamycin, a chemical ER stress inducer, in the absence or presence of GN for 12 h. As shown in Figure 1D, GN treatment significantly reduced tunicamycin-induced mRNA levels of ER stress markers. Taken together, these results indicate that GN inhibits CB1R-induced ER stress in HepG2 cells.

### 2.2. GN Improved CB1R-Mediated Inhibition of Insulin Signaling in HepG2 Cells

**Figure 2:**
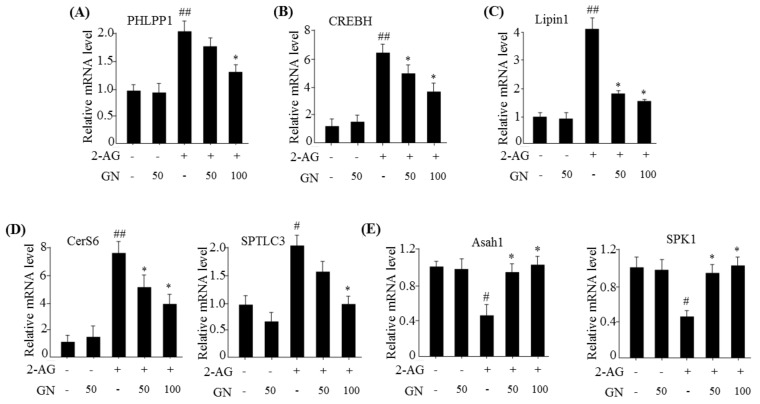
GN reversed 2-AG-mediated expression of insulin resistance-associated genes in HepG2 cells. HepG2 cells were incubated with 2-AG in the absence or presence of GN (50 or 100 µM) for 12 h. qPCR analysis of *PHLPP1* (**A**), *CREBH* (**B**), *Lipin1* (**C**), *Cer6* and *SPTLC3* (**D**), and *Asah1* and *SPK1* (**E**). Values are expressed as mean ± SEM (*n* = 3 independent experiments). ^#^ *p* < 0.05, ^##^ *p* < 0.01 vs. untreated control. * *p* < 0.05 vs. 2-AG-treated control.

Hepatic CB1R-induced ER stress contributes to insulin resistance by inhibiting insulin signaling via several ER stress-dependent mechanisms [3,4,5]. CB1R-induced ER stress stimulates the expression of serine/threonine phosphatase *PHLPP1* and CREBH-dependent *Lpin1*, which contribute to inhibit insulin signaling. Therefore, we investigated whether GN suppresses CB1R-induced expression of *PHLPP1*, *CREBH*, and *Lipin1* in 2-AG-treated HepG2 cells. Results of qPCR revealed that 2-AG treatment increased mRNA levels of *PHLPP1* (Figure 2A), *CREBH* (Figure 2B), and *Lipin1* (Figure 2C). However, GN treatment significantly reversed 2-AG-induced effects. Furthermore, it has been reported that CB1R suppresses insulin signaling via regulation of ceramide production, which involves the balance of de novo ceramide synthesis and degradation of ceramides [5]. Therefore, we examined whether GN reverses the effect of CB1R on the expression of de novo ceramide synthesis-associated genes and ceramide degradation-associated genes in HepG2 cells. Treatment with 2-AG increased the mRNA levels of de novo ceramide synthesis-associated genes such as ceramide synthase 6 (*CerS6*) and serine-palmitoyl transferase LC3 (*SPTLC3*) (Figure 2D), whereas reduced the mRNA levels of ceramide degradation-associated genes such as *N*-acylsphingosine amidohydrolase 1 (*Asah1*) and sphingosine kinase 1 (*SPK1*) (Figure 2E). However, GN treatment reversed 2-AG-mediated effects, suggesting that GN suppresses CB1R-induced ceramide production, which might contribute to improve insulin resistance.

**Figure 3:**
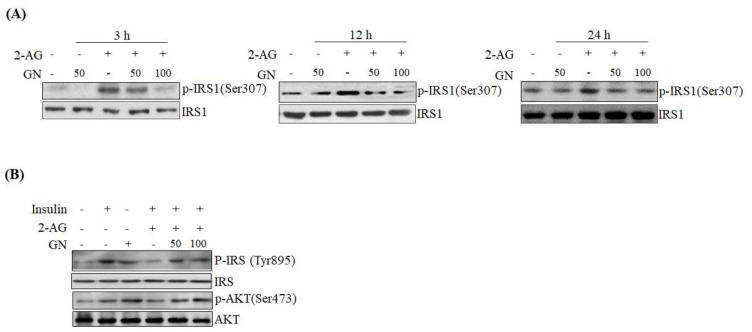
GN improved 2-AG-mediated inhibition of insulin signaling in HepG2 cells. (**A**) HepG2 cells were incubated with 2-AG in the absence or presence of GN (50 or 100 µM) for 3, 12 and 24 h. Serine-307 phosphorylation of IRS1 was detected by western blot analysis; (**B**) HepG2 cells were incubated with 2-AG in the absence or presence of GN (50 or 100 µM) for 12 h, and then incubated with insulin (10 nM) for 30 min. The phosphorylation of IRS1 (tyrosine-895) and AKT (serine-473) was detected by western blot analysis.

Then, we investigated the effects of GN on insulin signaling in 2AG-treated HepG2 cells. As shown in Figure 3A, 2-AG treatment increased the serine-307 phosphorylation of IRS1 at three different times, which inhibits insulin signaling; however, GN treatment reduced serine-307 phosphorylation of IRS1. Next, we investigated the effects of GN on insulin-activated phosphorylation of IRS1 at tyrosine 893 and AKT at serine 473 in 2-AG-treated HpG2 cells. As shown in Figure 3B, incubation with insulin resulted in increased phosphorylation of IRS1 (tyrosine-895) and AKT (serine-473), but 2-AG treatment reduced the expressions of p-IRS1 and p-AKT. However, GN treatment reversed the 2-AG-mediated reduction of insulin-induced phosphorylation of IRS1 and AKT (Figure 3B). These results indicate that GN improves hepatic CB1R-mediated inhibition of insulin signaling.

### 2.3. GN Inhibited CB1R-Induced Lipogenesis in HepG2 Cells

**Figure 4:**
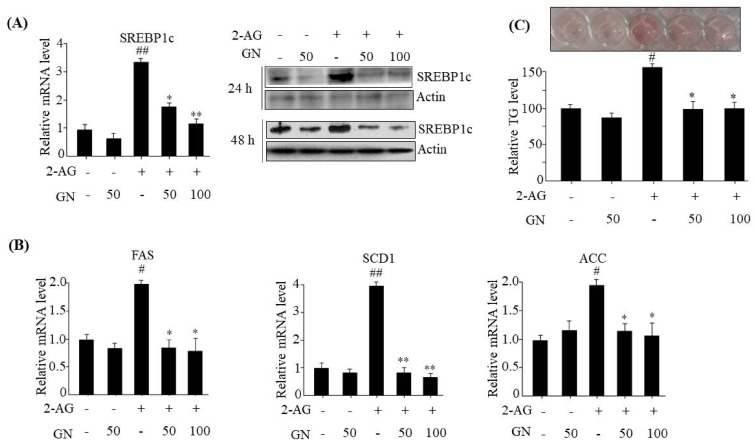
GN inhibited 2-AG-induced lipogenesis in HepG2 cells. HepG2 cells were incubated with 2-AG in the absence or presence of GN (50 or 100 µM) for 24 or 48 h. (**A**) qPCR and western blot analysis of SREBP1c; (**B**) qPCR analysis of *FSA*, *SCD1*, and *ACC*; (**C**) Intracellular TG levels were measured by TG measurement and ORO staining. Values are expressed as mean ± SEM (*n* = 3 independent experiments). ^#^ *p* < 0.05, ^##^ *p* < 0.01 vs. untreated control. * *p* < 0.05, ** *p* < 0.01 vs. 2-AG-treated control.

Activation of hepatic CB1R induces intracellular TG accumulation through upregulation of lipogenesis, which contributes to dysregulation of insulin signaling [6]. Therefore, we evaluated the inhibitory effects of GN on CB1R-induced lipogenesis in HepG2 cells. The expression of a key lipogenesis transcription factor SREBP1c and its downstream lipogenesis genes was measured in HepG2 cells after incubated with 2-AG in the absence or presence of different concentrations of GN for 24 h. As shown in Figure 4A, qPCR and western blot analyses showed that 2-AG treatment increased mRNA and protein levels of SREBP1c; however, GN reversed these changes. GN also reduced 2-AG-induced SREBP1c protein level even at longer incubation time (48 h). The mRNA levels of SREBP1c downstream lipogenesis genes including *FAS*, *SCD1*, and *ACC* were enhanced by 2-AG treatment, which were efficiently reversed by GN treatment (Figure 4B). In accordance with downregulation of lipogenesis genes, GN suppressed 2-AG-induced intracellular TG accumulation, as shown by TG measurement and ORO staining (Figure 4C).

### 2.4. GN Inhibited CB1R-Induced Gluconeogenesis in HepG2 Cells

**Figure 5:**
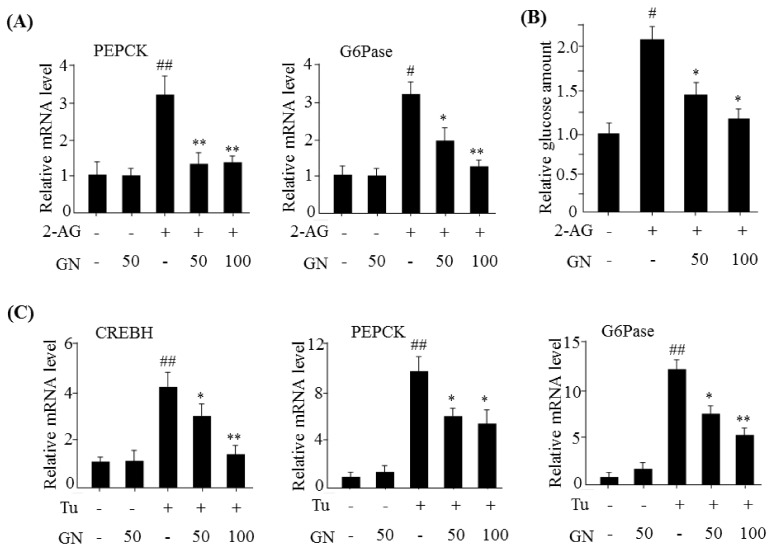
GN inhibited 2-AG-induced gluconeogenesis in HepG2 cells. (**A**) HepG2 cells were incubated with 2-AG in the absence or presence of GN (50 or 100 µM) for 12 h. qPCR analysis of *PEPCK* and *G6Pase*; (**B**) Measurement of glucose production; (**C**) HepG2 cells were incubated with tunicamycin (Tu) in the absence or presence of GN (50 or 100 µM) for 12 h. qPCR analysis of *CREBH*, *PEPCK*, and *G6Pase*. Values are expressed as mean ± SEM (*n* = 3 independent experiments). ^#^ *p* < 0.05, ^##^ *p* < 0.01 vs. untreated control. * *p* < 0.05, ** *p* < 0.01 vs. 2-AG or Tu-treated control.

The hepatic CB1R induces gluconeogenesis via upregulation of gluconeogenesis genes [7]. Therefore, we investigated inhibitory effects of GN on hepatic CB1R-induced gluconeogenesis in HepG2 cells. HepG2 cells were incubated with 2-AG in the absence or presence of different concentrations of GN for 12 h, and the expression of gluconeogenesis genes was measured by qPCR. As shown in Figure 5A, 2-AG treatment increased mRNA levels of gluconeogenesis genes such as phosphoenolpyruvate carboxykinase (*PEPCK*) and glucose 6-phosphatase (*G6Pase*); however, GN treatment reversed these changes. Then, we measured glucose production in 2-AG-treated HepG2 cells. Consistent with increased mRNA levels of gluconeogenesis genes, 2-AG treatment resulted in increased glucose production in HepG2 cells (Figure 5B). However, GN treatment markedly suppressed 2-AG-induced glucose production, indicating that GN inhibited CB1R-induced gluconeogenesis. To confirm whether GN-mediated inhibition of gluconeogenesis occurs via the suppression of ER stress, we investigated the inhibitory effects of GN on the expression of gluconeogenesis genes in HepG2 cells. HepG2 cells were incubated with tunicamycin, an ER stress inducer, in the absence or presence of different concentrations of GN for 12 h, and the expression of gluconeogenesis genes was measured by qPCR. As shown in Figure 5C, tunicamycin treatment increased the mRNA levels of *CREBH*, *PEPCK*, and *G6Pase*; however, GN reversed these changes. Taken together, these results suggest that GN might inhibit hepatic CB1R-induced gluconeogenesis via inhibition of ER stress in HepG2 cells.

### 2.5. GN Ameliorated HFD-Induced Hyperglycemia through Inhibition of Hepatic CB1R-Dependent Insulin Resistance and Gluconeogenesis

**Figure 6:**
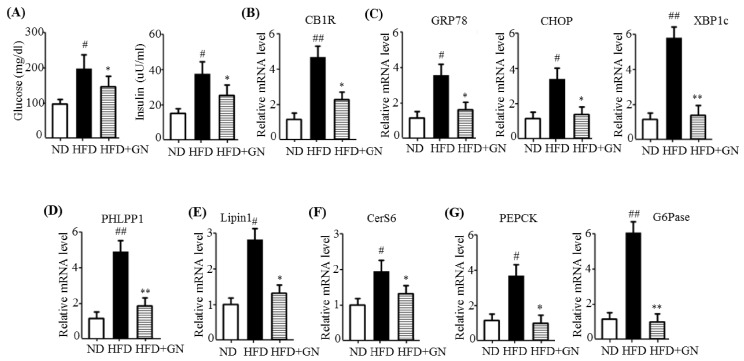
GN ameliorated high-fat diet (HFD)-induced hyperglycemia through inhibition of hepatic CB1R signaling. C57BL6 mice were fed HFD for 12 weeks and orally administered GN for 6 weeks. (**A**) Fasting levels of glucose and insulin. qPCR analysis of *CB1R* (**B**), *GRP78*, *CHOP*, and *XBP1c* (**C**), *PHLPP1* (**D**), *Lipin1* (**E**), *CerS6* (**F**), and *PEPCK* and *G6Pase* (**G**). The values are expressed as mean ± SEM (*n* = 5 mice per group). ^#^ *p* < 0.05, ^##^ *p* < 0.01 vs. ND mice. * *p* < 0.05, ** *p* < 0.01 vs. HFD-induced obese mice control. ND; normal diet.

It has been demonstrated that HFD impairs hepatic insulin signaling via CB1R activation, which contributes to insulin resistance and gluconeogenesis, resulting in hyperglycemia [3]. Thus, pharmacological inhibition of CB1R improves HFD-induced hyperglycemia and glucose tolerance. Previously, we demonstrated that GN reduced the HFD-induced hyperglycemia and improved the glucose tolerance in HFD obese mice [13]. In the current study, we examined whether GN-mediated improvement of hyperglycemia and glucose tolerance is through the inhibition of hepatic CB1R-mediated insulin resistance and gluconeogenesis. HFD induced obesity (Supplementary Figure S1). Consistent with previous results, we observed that GN reduced HFD-induced increase in the serum levels of glucose and insulin in HFD obese mice (Figure 6A). Subsequently, we evaluated the expression of CB1R, ER stress markers, insulin resistance-associated genes, and gluconeogenesis genes in the liver of HFD-induced obese mice. As shown in Figure 6, GN reversed the HFD-induced mRNA levels of *CB1R* (Figure 6B), ER stress markers such as *GRP78*, *CHOP*, and *XBP1c* (Figure 6C), *PHLPP1* (Figure 6D), *Lipin1* (Figure 6E), *CerS6* (Figure 6F), and gluconeogenesis genes such as *PEPCK* and *G6Pase* (Figure 6G). However, GN administration significantly reversed these HFD-induced effects.

**Figure 7:**
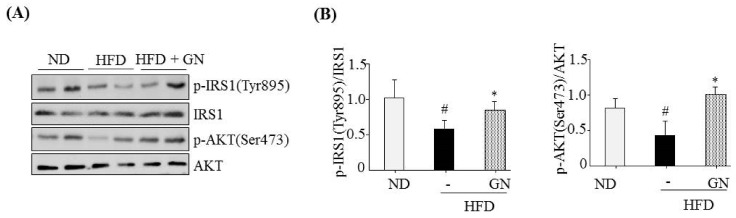
GN reversed HFD-mediated inhibition of insulin signaling in the liver of HFD obese mice. C57BL6 mice were fed HFD for 12 weeks and orally administered GN for 6 weeks. The liver homogenates were subjected to western blot using indicated antibody. (**A**) Representative western blotting. (**B**) Densitometric results. The values are expressed as mean ± SEM (*n* = 5 mice per group). *^#^ p* < 0.05 vs. ND mice. * p < 0.05 vs. HFD-induced obese mice control. ND; normal diet.

Finally, we investigated whether GN improved HFD-mediated inhibition of insulin signaling in the liver of HFD-induced obese mice. HFD feeding reduced both phosphorylation of IRS1 at tyrosine 895 and AKT at serine 473 (Figure 7); however, GN administration to HFD obese mice reversed HFD-mediated effects, suggesting that GN reversed the HFD-induced inhibition of insulin signaling in mice. Taken together, these results indicate that GN ameliorates HFD-induced insulin resistance and gluconeogenesis, which may play an important role in GN-mediated improvement of hyperglycemia.

## 3. Discussion

The activation of peripheral CB1Rs has been increasingly recognized as an important regulator of metabolic disorders due to deleterious effects on lipid and glucose metabolism [2]. Among peripheral CB1Rs, activation of the hepatic CB1R induces ER stress-dependent hepatic insulin resistance and gluconeogenesis, resulting in hyperglycemia [3,7]. In addition, hepatic CB1R causes lipid accumulation by upregulation of lipogenesis genes, resulting in hepatic steatosis [6]. Therefore, the inhibition of hepatic CB1R signaling is a promising target for treating type 2 diabetes and hepatic steatosis. GN is a phytochemical derived from *S. chinensis*, a traditional medicinal herb [11]. Previously, we demonstrated that GN exerts protective effects against HFD-induced hyperglycemia and hepatic steatosis in HFD obese mice [12,13,14]. In the present study, we investigated whether GN-mediated improvement of hyperglycemia and hepatic steatosis is through inhibition of the hepatic CB1R signaling. We examined the inhibitory effect of GN on CB1R-induced insulin resistance and gluconeogenesis in vitro and in vivo.

Recent studies have demonstrated that obesity leads to activation of the hepatic CB1R signaling, which contributes to insulin resistance and gluconeogenesis in the ER stress dependent manner, resulting in hyperglycemia [3,4,5,6,7]. Therefore, hepatic CB1R-mediated ER stress can be a potential target for HFD-induced hyperglycemia. Previously, we demonstrated that GN inhibited fatty acid-induced ER stress and prevented HFD-induced hepatic steatosis and hyperglycemia [12,13]. In the current study, we investigated whether GN also inhibited CB1R-induced ER stress and subsequently improved CB1R-mediated insulin resistance and gluconeogenesis in HepG2 cells. Our data showed that treatment with 2-AG, a CB1R activator, promoted the expression of ER stress markers such as *GRP78*, *CHOP*, and *XBP1c* in HepG2 cells, but GN significantly prevented 2-AG-induced expression of these genes, indicating that GN inhibits CB1R-induced ER stress in HepG2 cells.

It has been reported that CB1R-induced ER stress causes insulin resistance via several mechanisms [3,4,5,6]. As described previously, hepatic CB1R-induced ER stress leads to insulin resistance by upregulation of serine phosphatase PHLPP1, Lipin1, and ceramide production. PHLPP1 reverses insulin-activated AKT phosphorylation and inhibits insulin signaling. Lipin1 generates DAG, which subsequently suppresses insulin signaling via PKC activation. Therefore, we examined the inhibitory effects of GN on the expression of *PHLPP1* and *Lipin1* in 2-AG-treated HepG2 cells. Our data revealed that GN treatment significantly reversed 2-AG-induced mRNA levels of both *PHLPP1* and *Lipin1*. CB1R-induced ceramide production, which is regulated by de novo synthesis and degradation of ceramide, also leads to inhibition of insulin signaling. To investigate whether GN affects CB1R-induced ceramide production, we examined the expression of de novo ceramide synthesis genes and ceramide degradation genes in 2-AG-treated HepG2 cells. GN reduced the 2-AG-induced mRNA expression of *CerS6* and *SPTLC3* involved in *de novo* ceramide synthesis, but increased the expression of mRNA levels of *Asah1* and *SPK1* involved in ceramide degradation, which were decreased by 2-AG treatment, indicating that GN suppresses CB1R-induced ceramide production. Taken together, these results suggest that GN might contribute to improvement of hepatic insulin resistance by inhibition of CB1R-induced PHLPP1, Lipin1, and ceramide production. To confirm the ameliorative effect of GN on insulin resistance, we examined insulin signaling in 2-AG-treated HepG2 cells. Treatment with 2-AG increased serine-307 phosphorylation of IRS1, which inhibits AKT phosphorylation, and resulted in reduced insulin-stimulated phosphorylation of IRS1 (tyrosine-895) and AKT (serine-473); however, GN reduced the phosphorylation of IRS1 (serine-307), whereas increased the insulin-stimulated phosphorylation of IRS1 (tyrosine-895) and AKT (serine-473). These results demonstrated that GN reverses the hepatic CB1R-mediated inhibition of insulin signaling. 

The hepatic CB1R has been reported to induce TG accumulation via upregulation of lipogenesis genes expression, which leads to dysregulation of insulin signaling [6]. Thus, we tested whether GN inhibits CB1R-induced lipogenesis in 2-AG-treated HepG2 cells. We found that GN inhibited 2-AG-induced expression of lipogenesis genes including *SREBP1c*, *FAS*, *SCD1*, *ACC*, and subsequent intracellular TG accumulation in HepG2 cells. These results suggest that prevention of TG accumulation might also play an important role in GN-mediated improvement of insulin resistance and hepatic steatosis.

The hepatic CB1R stimulates gluconeogenesis via induction of ER stress, which plays a role in HFD-induced hyperglycemia [7]. CB1R-induced ER stress stimulates the expression of gluconeogenesis genes via CREBH. Thus, we investigated the inhibitory effect of GN on gluconeogenesis in 2-AG-treated HepG2 cells. Treatment with 2-AG promoted the expression of *CREBH*, *PEPCK*, and *G6Pase*, and resulted in increased glucose production. However, GN inhibited 2-AG-induced expression of gluconeogenesis genes and subsequent glucose production in HepG2 cells. To confirm whether GN-mediated inhibition of gluconeogenesis is through suppression of ER stress, we assessed the inhibitory effects of GN on the expression of gluconeogenesis genes in tunicamycin-treated HepG2 cells. Consistent with the results in 2-AG-treated HepG2 cells, GN treatment reversed tunicamycin-induced expression of gluconeogenesis genes including *CREBH*, *PEPCK*, and *G6Pase*. Taken together, these results indicate that GN inhibits CB1R-induced gluconeogenesis via suppression of ER stress, which might contribute to improvement of hyperglycemia.

HFD activates hepatic CB1R signaling and results in insulin resistance and hyperglycemia [3]. Our previous study revealed that GN administration to HFD obese mice efficiently reduced HFD-induced hyperglycemia, and improved glucose tolerance in mice [13]. Thus, in the current study, we investigated whether the inhibition of hepatic CB1R signaling plays a role in GN-mediated improvement of hyperglycemia and glucose tolerance. We assessed the inhibitory effects of GN on CB1R-mediated insulin resistance and gluconeogenesis in the liver of HFD obese mice. Results showed that GN administration to HFD obese mice reversed HFD-induced expression of *CB1R*, ER stress markers, *PHLPP1*, ceramide synthase *CerS6*, and recovered reduced phosphorylation of IRS-1 at tyrosine 895 and AKT at serine 473 in the livers of HFD obese mice. Furthermore, GN efficiently reduced HFD-induced expression of gluconeogenesis genes, *PEPCK* and *G6Pase*. These results indicate that GN can ameliorate HFD-induced hyperglycemia through inhibition of CB1R-induced insulin resistance and gluconeogenesis.

Activation of CB1R signaling in the CNS increases food intake and induces obesity [15]. Rimonabant, an inverse agonist of central CB1R, was used as an anti-obesity drug [16]. However, it was withdrawn from the market because of its psychiatric side effects. Since the withdrawal of rimonabant, many investigators have studied peripheral CB1R inhibitors with lesser side effects. Natural products derived from medicinal herbs are usually considered less toxic with fewer side effects. GN is active component of *S. chinensis* that has been used as a traditional herbal medicine and has diverse pharmacological activities [9]. Our results suggest that GN is an attractive and potent compound that inhibits peripheral CB1R signaling and can be useful in the treatment of metabolic disorders including type 2 diabetes.

In conclusion, GN inhibits CB1R-induced ER stress and results in improvement of insulin resistance and gluconeogenesis, which might contribute to the amelioration of hyperglycemia.

## 4. Materials and Methods

### 4.1. Reagents

GN (≥98% purity) was obtained from ChemFaces (Wuhan, China). Tunicamycin and 2-AG were purchased from Sigma-Aldrich (St. Louis, MO, USA). Dulbecco’s modified Eagle’s medium (DMEM), penicillin–streptomycin, and fetal bovine serum (FBS) were obtained from Gibco BRL (Grand Island, NY, USA). Antibodies against GRP78, CHOP, and XBP1c were purchased from Santa Cruz Biotechnology (Santa Cruz, CA, USA). Antibodies against p-IRS1 (serine-307), p-IRS1 (tyrosine-895), p-AKT (serine-473), IRS1, and AKT were purchased from Cellular Signaling Technology (Danvers, MA, USA).

### 4.2. Cell Culture

The human hepatocellular carcinoma cell line HepG2 was obtained from the American Type Culture Collection (Manassas, VA, USA). HepG2 cells were cultured in DMEM supplemented with 10% heat-inactivated fetal bovine serum, 20 U/mL penicillin, and 20 μg/mL streptomycin.

### 4.3. Quantitative Polymerase Chain Reaction (qPCR)

Total RNA was isolated from HepG2 cells and mouse livers using TRIzol^TM^ (Invitrogen, Darmstadt, Germany), as per manufacturer’s instructions. One microgram of the isolated RNA was reverse-transcribed by using TOPScript RT DryMix (Enzynomics, Daejeon, Korea). Quantitative real-time PCR was performed using a SYBR Green premixed Taq reaction mixture with gene-specific primers. The gene-specific primers used in this study are listed in Supplementary Table S1.

### 4.4. Western Blots

Proteins (40 μg per well) were separated from HepG2 cells using SDS-PAGE on 8% gels and transferred to polyvinylidene fluoride membranes. The membranes were incubated with primary antibodies, followed by incubation with anti-rabbit or anti-mouse secondary antibodies (Santa Cruz Biotechnology, Dallas, TX, USA) and protein bands were visualized using an enhanced chemiluminescence system (ECL Advance, GE Healthcare, Hatfield, UK).

### 4.5. Triglyceride (TG) Measurement

HepG2 cell suspensions were mixed with 750 μL of chloroform/methanol/H2O (8:4:3, *v*/*v*/*v*) to extract TG. The cell suspensions were incubated at room temperature for 1 h and centrifuged at 800× *g* for 10 min. The bottom layer (organic phage) obtained was dried overnight and then dissolved in ethanol, followed by measurement of TG concentrations using an AM 157S-K TG kit (Asan Pharmaceutical, Seoul, Korea), which was normalized to the protein concentration.

### 4.6. Oil Red O Staining

HepG2 were washed twice with phosphate-buffered saline (PBS) and fixed with 10% formalin for 60 min. And then, the cells were then stained with an Oil Red O (ORO) working solution (1.5 mg/mL ORO/60% isopropanol) for 60 min at room temperature. After staining, the cells were washed with distilled water and photographed under a light microscope.

### 4.7. Glucose Production

Glucose production from HepG2 cells was measured using a colorimetric glucose oxidase assay according to the manufacturer’s protocol (Sigma-Aldrich). Briefly, cells were washed three times with PBS and incubated in glucose production buffer (glucose-free DMEM (pH 7.4), 20 mM sodium lactate, 1 mM sodium pyruvate, and 15 mM HEPES) for 3 h at 37 °C in 5% CO_2_. Glucose concentration was normalized to cellular protein concentration.

### 4.8. Animal Study

C57BL/6 mice (male, 6-week-old) were purchased from Jung-Ang Lab Animal, Inc. (Seoul, Korea). The animals were housed in standard conditions of temperature (21–23 °C), humidity (40–60%), and a 12-h light/dark cycle, and were given free access to food and water. The mice were fed a normal diet (ND) or an HFD for 12 weeks. Subsequently, the HFD-fed mice were divided into the following two groups (*n* = 6 per group): HFD (distilled water-treated) group or HFD + GN (20 mg/kg of body weight) group. The experimental diets were TD.06414, a high-fat in which 60% calories are from fats, and the control diet in which 10% calories are from fat. GN was administered orally every day for 6 weeks. The animal protocol used in this study was reviewed and approved by the Pusan National University’s Institutional Animal Care and Use Committee in accordance with established ethical and scientific care procedures (approval number: PNU-2017–1456; 7 February 2017).

### 4.9. Biochemical Analysis

After starvation for 12 h, the mice were sacrificed. The blood samples were collected and centrifuged at 1000× *g* for 15 min at 4 °C to obtain serum, which was stored at −80°C until analysis. The concentrations of blood glucose and insulin were determined by using commercial analysis kits (Asan Pharmaceutical, Seoul, Korea).

### 4.10. Statistical Analysis

All data are presented as the mean ± standard error of the mean (SEM). The statistical differences between various groups were examined by one-way analysis of variance (ANOVA) followed by Tukey’s test. Values with *p* < 0.05 were considered statistically significant.

## Supplementary Materials

Supplementary materials can be found at http://www.mdpi.com/1422-0067/19/4/968/s1.

## Abbreviations

2-AG	2-arachidonoylglycerol
Asah1	*N*-acylsphingosine amidohydrolase1
CB1R	cannabinoid type 1 receptor
CerS6	ceramide synthase 6
CREBH	cAMP-responsive element-binding protein H
CHOP	C/EBP homologous protein
ER	endoplasmic reticulum
G6Pase	glucose 6-phosphatase
GN	gomisin N
GRP78	glucose-regulated protein 78
HFD	high fat diet
PEPCK	phosphoenolpyruvate carboxykinase
PHLPP1	PH domain leucine-rich repeats protein phosphatase 1
SPK1	sphingosine kinase 1
SPTLC3	serine-palmitonyl transferase LC3
XBP1c	X box-binding protein 1, C-terminal
