# Supplementary material for: Protective Effects of Gomisin N against Hepatic Cannabinoid Type 1 Receptor-Induced Insulin Resistance and Gluconeogenesis
Source: Int J Mol Sci. 2018 Mar 23;19(4):968. doi: 10.3390/ijms19040968 (PMC5979504; doi:10.3390/ijms19040968)
Supplement: Supplementary file 1 [file ijms-19-00968-s001.pdf]

Supplementary

Protective Effects of Gomisin N against Hepatic Cannabinoid Type 1 Receptor-Induced Insulin Resistance and Gluconeogenesis

Arulkumar Nagappan, Dae Young Jung, Ji-Hyun Kim and Myeong Ho Jung

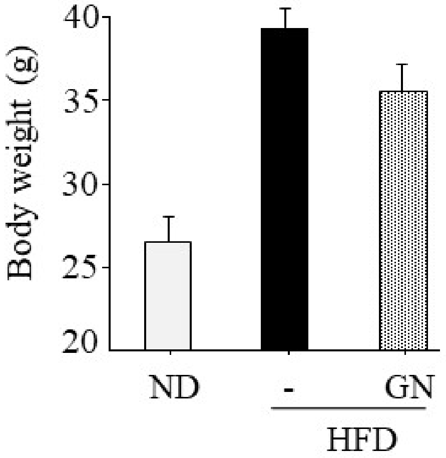

Figure S1. Body weight.

Table S1. List of primers for qPCR.

Gene	Forward Primer	Reverse Primer
hCB1R	5′-AAGGTGACATGGCATCCAAAT-3′	5′-AGGACGAGAGAGACTTGTTGTA-3′
hPHLPP1	5′-AGGCGCATGCACACCGTGAT-3′	5′-GGACAAGGCGCGGGTTTCCA-3′
hLipin1	5′-TGCTGGAGAGCAGCAGAACTC-3′	5′-TAGGGTATGAGGCTGACTGAG-3′
hAsah1	5′-GCATCAACACAGGAGAGTC-3′	5′-GGAGGCAGAGGCATAGAG-3′
hCerS6	5′-ACATTCCTTCAGCCTCCTGGAGTT-3′	5′-GCTCCCTGGTTTCCAGGCCAC-3′
hSPTLC3	5′-TATTCCCGGCACAAGAAGTC-3'	5′-CTGAGGAAGGGGGAGAAATC-3′
hSPK1	5′-ATGCTGGCTATGAGCAGGTC-3′	5′-GTGCAGAGACAGCAGGTTCA-3′
hCREBH	5′-CGGATTTAGCTGCTGGAAAG-3′	5′-AGCTCCACGTGTCTCAGGAT-3′
hGRP78	5′-ATGATGCTGAGAAGTTTGCTGA-3′	5′-GGAAAGTTTACCTCCCAGCTTT-3′
hCHOP	5′-AGGGAGAACCAGGAAACGGAA AC-3′	5′-TCCTGCTTGAGCCGTTCATTCT-3′
hXBP1c	5′-TGCTGAGTCCGCAGCAGGTG-3′	5′-GCTGGCAGGCTCTGGGGAAG-3′
hG6Pase	5′-GGGAAAGATAAAGCCGACCTAC-3′	5′-CAGCAAGGTAGATTCGTGACA-3′
hPEPCK	5′-TGACAACTGCTGGTTGGCT-3′	5′-TGGTGCGACCTTTCATGC-3′
mCB1R	5′-AAGTCGATCTTAGACGGCCTT-3′	5′-TCCTAATTTGGATGCCATGTCT-3′
mPHLPP1	5′-CTGGCGTGATAGCGGGCGAG-3′	5′-CCAGGCGCCGGGTAGTCTCT-3′
mLipin1	5′-CCCTCGATTTCAACGTACCC -3′	5′-GCAGCCTGTGGCAATTCA-3′
mCerS6	5′-GCTGGTTTCGACAAAGACG-3′	5′-AGAGGTAAAAGGAAAATCTCCA-3′
mGRP78	5′-GAAAGGATGGTTAATGATGCTGAG-3′	5′-GTCTTCAATGTCCGCATCCTG-3′
mCHOP	5′-CAGTCATGGCAGCTGAGTCC-3′	5′-TAGGTGCCCCCAATTTCATC-3′
mXBP1c	5′-GAG TCC GCA GCA GGT G-3′	5′-GTG TCA GAG TCC ATG GGA-3′
mG6Pase	5′-AAGCCAACGTATGGATTCCG-3′	5′-ACAGCAATGCCTGACAAGACT-3′
mPEPCK	5′-TCTCTGATCCAGACCTTCCAA-3′	5′-GAAGTCCAGACCGTTATGCAG-3′
